# Laser and Energy-Based Devices in Aesthetic Practice: A 10-Year Single-Surgeon Review of Treatments, Complications, and Same-Practice Surgical Crossover

**DOI:** 10.1093/asjof/ojag105

**Published:** 2026-06-11

**Authors:** Carolyn Kim, Bhavana Thota, Vidhya Nadarajan, Anca Dogaroiu, Lauren Kim, Amor Niksic, Victoria Peters, Jennifer Barillas, John E Hoopman, Jeffrey M Kenkel

## Abstract

**Background:**

The use of lasers and energy-based devices in aesthetic plastic surgery has increased substantially; yet, long-term treatment patterns and their relationship to subsequent surgery remain unclear.

**Objectives:**

To evaluate patient demographics, device utilization patterns, complications, and same-practice surgical crossover over a 10-year single plastic surgeon's experience.

**Methods:**

A retrospective review was conducted of all patients who underwent laser or energy-based treatments by the senior author between January 1, 2013 and January 1, 2023. Variables included patient demographics, device type, procedural characteristics, documented complications, and subsequent aesthetic procedures performed by the same surgeon. Treatment patterns were evaluated longitudinally by comparing device utilization at the initial, third, and final visits.

**Results:**

A total of 632 patients underwent 1905 laser or energy-based treatments. Patients were predominantly female (85.8%) with a mean age of 52.8 years at initial presentation. Fitzpatrick skin types II-III were the most common (72.2%). Intense pulsed light was the most frequently used modality overall (47.6%) and at the initial visit (40.3%), with increasing use of ablative modalities over time. A total of 26 complications (1.4%) were documented, most commonly hyperpigmentation (n = 7). Overall, 40.5% of patients underwent subsequent aesthetic surgery, accounting for 523 procedures, with a mean time to surgery of 4.2 years.

**Conclusions:**

Lasers and energy-based treatments demonstrated a low rate of documented complications in single-surgeon practice and showed evolving patterns over time. A substantial proportion of patients underwent subsequent aesthetic surgery within the same practice, suggesting these modalities may play an important role in longitudinal patient engagement and the integration of nonsurgical and surgical care within comprehensive aesthetic care.

**Level of Evidence: 4 (Therapeutic):**

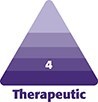

The use of lasers and other energy-based devices in aesthetic plastic surgery has increased substantially over the past decade.^1-3^ According to The Aesthetic Society's *Aesthetic Plastic Surgery National Databank Statistics*, 1,047,794 combination laser skin treatments and 2,758,690 total energy-based procedures were performed in 2022, generating $776,058,800 and $1,540,845,902 in revenue, respectively.^4^ These treatments can be performed independently or as adjuncts to aesthetic surgery to address a range of cosmetic concerns, including photodamage, dyspigmentation, ultraviolet spots, brown spots, fine lines, wrinkles, and textural irregularities.^5-10^ The versatility of lasers stems in part from their ability to deliver controlled energy to specific chromophores, enabling selective photothermolysis and precise targeting within tissue. Since their introduction into clinical practice in the 1980s, continual technological advances have improved energy delivery, target specificity, and overall treatment safety.^11-13^

Ablative lasers, such as the 10,600-nm carbon dioxide (CO_2_) laser and the 2940-nm erbium:yttrium-aluminium-garnet (Er:YAG) laser, vaporize the epidermal and superficial dermal layers of the skin, resulting in more aggressive resurfacing and more pronounced clinical improvements.^14,15^ However, these modalities are associated with increased patient discomfort and longer recovery times.^14,15^ To reduce downtime and minimize side effects, nonablative lasers (532-nm, 1064-nm, 1470-nm, and 1927-nm wavelengths) were developed. These devices induce controlled thermal injury to the subepidermal layer while preserving the superficial epidermis, promoting faster recovery though often yielding more modest results.^14,16^ Fractional resurfacing technologies, such as the 2910-nm fractionated laser, attempt to bridge this gap by creating microscopic columns of thermal ablation in the epidermis and dermis in a controlled, regularly spaced array.^17^ Hybrid systems, such as the dual-wavelength 1470/2940-nm laser, further integrate the delivery of both nonablative and ablative wavelengths to the same microscopic treatment zone to maximize benefit within a single pass.^10^

In addition to lasers, intense pulsed light (IPL) delivers noncoherent, polychromatic light with wavelengths ranging from 400 to 1200 nm. Filters may be used to preferentially target certain chromophores, such as hemoglobin or melanin, offering versatile treatment options for vascular and pigmentary lesions.^6,14,18^ Additional energy-based devices, including radiofrequency systems, further expand nonsurgical rejuvenation by heating the dermis to stimulate neocollagenesis without generating plume or epidermal ablation.^14,19,20^

Prior studies suggest that nonsurgical facial rejuvenation procedures, including neuromodulators and soft tissue fillers, may serve as an entry point into aesthetic medicine, with a subset of patients later pursuing surgical intervention.^21-23^ Whether similar patterns exist among patients undergoing laser or other energy-based treatments remains unclear. Given the increasing use and broad applicability of these technologies, evaluating patient utilization patterns and subsequent crossover to surgical procedures may provide insight into longitudinal aesthetic practice dynamics. Thus, this study aims to characterize patient demographics, laser and energy-based device utilization, complications, and same-practice surgical crossover within a single-plastic surgeon cohort over a 10-year period, providing insight into the role of these modalities within the continuum of comprehensive aesthetic care.

## METHODS

### Study Population and Design

**Figure 1.**
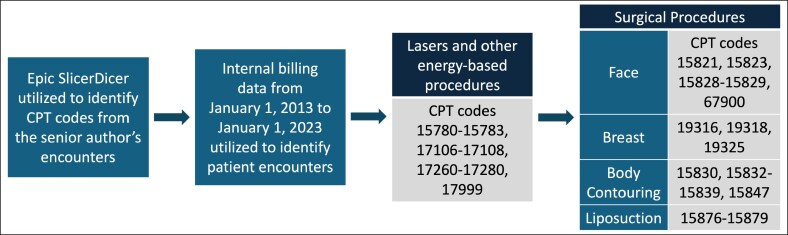
Flow diagram depicting study methodology. CPT, current procedural terminology.

This retrospective study included all patients aged 18 years or older who underwent a laser or energy-based procedure performed by the senior author at a single, high-volume academic center between January 1, 2013 and January 1, 2023. Institutional Review Board approval was obtained prior to data collection. Using an electronic medical record querying tool (SlicerDicer, Epic Systems, Verona, WI) and departmental billing records, 632 patients undergoing a total of 1905 procedures were identified. Relevant CPT codes were used to capture all laser and energy-based modalities, including IPL, nonablative and ablative lasers, and radiofrequency technologies, as well as any subsequent aesthetic facial, breast, body contouring, and liposuction procedures performed by the same surgeon (Figure 1). Patients treated by licensed aestheticians at the study institution were not included in this analysis.

### Data Collection and Statistical Analysis

Collected data included patient demographics, device type, procedural details, documented complications, and characteristics of subsequent aesthetic surgical cases. Devices captured in this study included IPL (mJOULE platform with a Broadband Light, Sciton Inc., Palo Alto, CA), 532/1064-nm laser (excel V, Cutera, Brisbane, CA), 1470/2940-nm laser (HALO, Sciton Inc.), 1927-nm laser (MOXI, Sciton Inc.), 2910-nm laser (UltraClear, Acclaro Medical, Smithfield, RI), 2940-nm Er:YAG laser (Sciton Inc.), 10,600-nm CO_2_ laser (UltraPulse, Lumenis, Yokneam, Israel), and various radiofrequency systems.

Primary endpoints included complication incidence and the rate of crossover to surgical intervention. Complications were identified through retrospective chart review of clinical documentation; however, mild or transient effects, particularly postinflammatory hyperpigmentation, were inconsistently documented and therefore likely underreported. Dates of all procedures were recorded to assess temporal relationships among laser and energy-based treatments and subsequent aesthetic surgery. Additionally, to evaluate changes in treatment patterns over time, device type was analyzed at three time points: the initial visit (all patients), the third visit (among patients with ≥3 visits, selected to approximate the cohort's mean number of encounters of 2.68), and the final recorded visit (among patients with >3 visits). Descriptive statistical analyses were performed using Microsoft Excel (version 16.77, Redmond, WA).

## RESULTS

**Figure 2.**
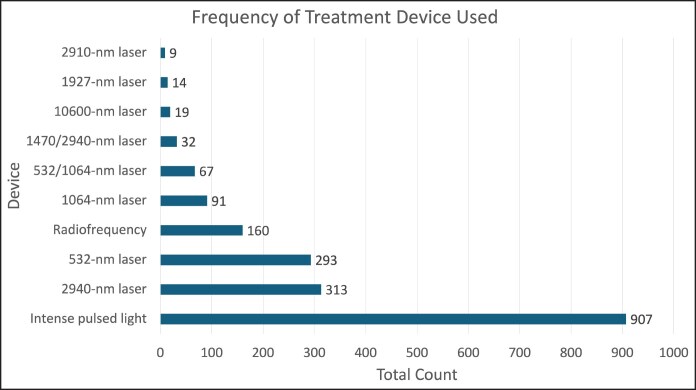
Frequency of laser and energy-based devices used by the senior author from January 2013 to January 2023.

From January 2013 to January 2023, 632 patients underwent a total of 1905 laser or energy-based procedures performed by the senior author. Patients were predominantly female (85.8%, *n* = 542) with a mean age of 52.8 ± 13.9 years at initial presentation (Table 1). Most patients had Fitzpatrick skin types II-III (72.2%, *n* = 456). Nearly half of patients had a single visit (45.1%, *n* = 285), followed by 2 visits (22.6%, *n* = 143), 3 visits (11.4%, *n* = 72), and 4 to 5 visits (11.4%, *n* = 72) for laser or energy-based treatments (Table 2). One patient had a maximum of 35 encounters over the 10-year study period. Overall, IPL was the most frequently performed treatment (47.6%, *n* = 907), followed by 2940-nm Er:YAG laser (16.4%, *n* = 313) and 532-nm laser (15.4%, *n* = 293) (Figure 2).

**Table 1.** Patient Demographics

Characteristics	*n* = 632 patients
Age at first encounter, mean (SD)	52.8 years (13.9)
Sex, *n* (%)	
Female	542 (85.76%)
Male	90 (14.24%)
Fitzpatrick skin type, *n* (%)	
I	15 (2.37%)
II	225 (35.60%)
III	231 (36.55%)
IV	59 (9.34%)
V	32 (5.06%)
VI	5 (0.79%)
Unspecified	65 (10.28%)

SD, standard deviation.

**Table 2.** Frequency of Visits With the Senior Author for Laser or Energy-Based Treatments

Total number of visits	Patients, *n* (%)
1	285 (45.09%)
2	143 (22.63%)
3	72 (11.39%)
4-5	72 (11.39%)
6-10	44 (6.96%)
11-20	12 (1.90%)
21-25	2 (0.32%)
>25	2 (0.32%)

### Complications

A total of 26 complications were recorded across the 1905 procedures, yielding a per-procedure rate of 1.4%. Hyperpigmentation (*n* = 7) was the most commonly documented adverse event, followed by hypopigmentation (*n* = 6), scarring (*n* = 6), and transient neuropathic pain (*n* = 5) (Table 3). Hyperpigmentation occurred across a range of Fitzpatrick skin types (II-VI), though it was more frequently observed in skin types IV-VI (57.1%, *n* = 4). Hypopigmentation and scarring occurred exclusively following full-face 2940-nm Er:YAG resurfacing. When assessed by device, most complications were associated with 2940-nm Er:YAG treatments (76.9%, *n* = 20), with fewer events observed following IPL with a 515-nm filter (*n* = 2), 532-nm laser (*n* = 2), 1064-nm laser (*n* = 1), and radiofrequency (*n* = 1).

**Table 3.** Documented Complications and Per-patient Incidence Following Laser or Energy-Based Treatments

Complication	Patients, *n* (%)
Hyperpigmentation	7 (1.11%)
Hypopigmentation	6 (0.95%)
Scarring	6 (0.95%)
Neuropathic pain	5 (0.79%)
Herpetic outbreak	1 (0.16%)
Bell's palsy	1 (0.16%)

### Treatment Patterns Over Time

**Figure 3.**
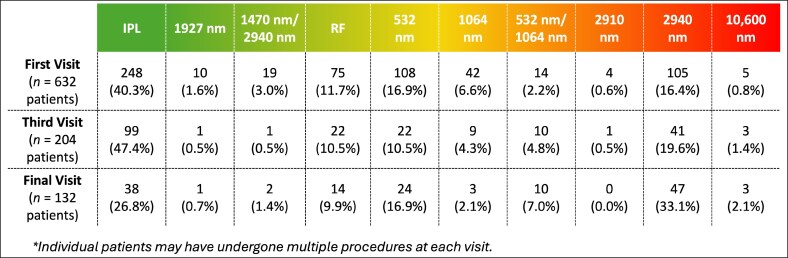
Frequency of laser and energy-based procedures over time from patients' initial, third, and final visits. IPL, intense pulsed light.

The most frequently performed treatment at the initial visit was IPL, comprising 40.3% (*n* = 248) of procedures (Figure 3). Among patients with multiple visits, there was an increasing proportion of ablative laser treatments over time. Specifically, use of the 2940-nm Er:YAG laser increased from 16.4% at the initial encounter to 33.1% at the final visit, while use of the 10,600-nm CO_2_ laser increased from 0.8% to 2.1%.

### Surgical Crossover

**Figure 4.**
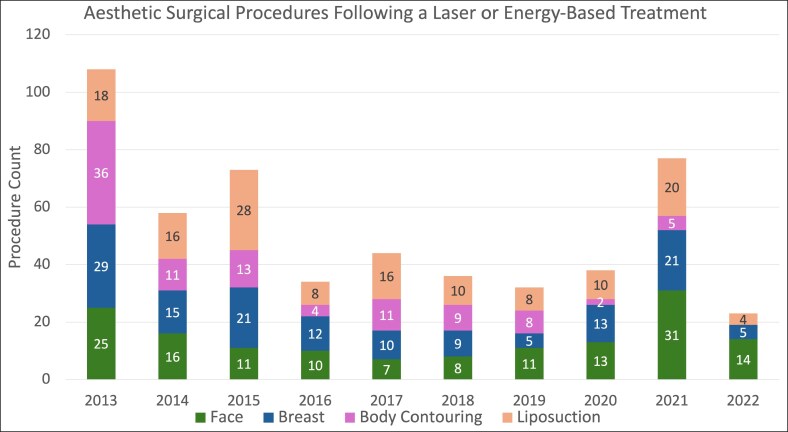
Trends in frequency and category of aesthetic surgical procedures among patients who initially underwent a laser or energy-based treatment.

A total of 256 patients (40.5%) underwent subsequent aesthetic surgery with the same surgeon following their laser or energy-based treatment, accounting for 523 procedures as some patients underwent multiple operations. The mean time from initial laser or energy-based treatment to surgery was 4.2 ± 3.3 years. Surgical procedures spanned facial (27.9%, *n* = 146), breast (26.8%, *n* = 140), body contouring (18.9%, *n* = 99), and liposuction (26.4%, *n* = 138) categories (Table 4, Figure 4). Overall, the most common operations were trunk liposuction (17.2%, *n* = 90), rhytidectomy (11.9%, *n* = 62), mastopexy (11.7%, *n* = 61), breast augmentation (11.5%, *n* = 60), and upper blepharoplasty (7.8%, *n* = 41).

**Table 4.** Aesthetic Surgical Procedures (n = 523) Performed in 256 Patients Following Laser or Energy-Based Treatments

Surgical procedure (CPT code)	Procedure count (*n*)	Percentage (%)
Face	146	27.92%
Blepharoplasty, lower eyelid (15821)	28	5.35%
Blepharoplasty, upper eyelid (15823)	41	7.84%
Rhytidectomy with SMAS flap (15829)	62	11.85%
Repair of brow ptosis (67900)	15	2.87%
Breast	140	26.77%
Mastopexy (19316)	61	11.66%
Reduction mammoplasty (19318)	19	3.63%
Breast augmentation with implant (19325)	60	11.47%
Body contouring	99	18.93%
Panniculectomy (15830)	11	2.10%
Excision of excessive skin and subcutaneous tissue, thigh (15832)	22	4.21%
Excision of excessive skin and subcutaneous tissue, hip (15834)	16	3.06%
Excision of excessive skin and subcutaneous tissue, arm (15836)	25	4.78%
Excision of excessive skin and subcutaneous tissue, other (15839)	16	3.06%
Abdominoplasty (15847)	9	1.72%
Liposuction	138	26.39%
Liposuction, head and neck region (15876)	21	4.02%
Liposuction, trunk (15877)	90	17.21%
Liposuction, upper extremity (15878)	6	1.15%
Liposuction, lower extremity (15879)	21	4.02%

Individual patients may have undergone multiple procedures.

CPT, current procedural terminology.

## DISCUSSION

The growing popularity of laser and energy-based technologies parallels advances in device safety, versatility, and clinical efficacy since their introduction in the 1980s.^4^ Their application now extends across multiple surgical and medical specialties, including gynecology, ophthalmology, otolaryngology, urology, and more.^24-27^ This study provides a descriptive analysis of laser and energy-based device utilization, complications, and same-practice surgical crossover within a single plastic surgeon's academic practice over a 10-year period, offering insight into real-world treatment patterns and longitudinal patient engagement.

In aesthetic plastic surgery, IPL remains one of the most commonly used modalities due to its broad wavelength spectrum, ability to target multiple chromophores, and limited downtime.^28,29^ This was reflected in our findings, where IPL accounted for nearly half of all light and energy-based treatments delivered by the senior author and was also the most frequently utilized initial treatment, supporting its role as an approachable entry point for patients who may be unfamiliar with other modalities. Our longitudinal analysis also showed an increasing proportion of ablative treatments over time, with greater utilization of the 2940-nm Er:YAG and 10,600-nm CO_2_ lasers at later visits. This increase may reflect evolving surgeon experience and practice patterns over the study period. It may additionally suggest a stepwise treatment progression, in which patients elect to undergo more intensive procedures as they gain familiarity with available technologies, develop trust in their provider, and refine their aesthetic goals. However, given the retrospective nature of this study, patient motivations could not be directly assessed, and prospective studies are needed to better characterize decision-making factors and motivations.

### Complications

Known adverse effects of laser and energy-based procedures include pigmentary changes, blistering, scarring, prolonged erythema, edema, ecchymoses, and burns.^28^ Among these, postinflammatory hyperpigmentation is the most common and is particularly observed in patients with higher Fitzpatrick skin types (IV-VI), which was consistent with our findings.^30,31^ Reported complication rates vary across the literature, with one prospective study by Batra and colleagues reporting up to 25% of patients developed pigmentation irregularities, of which 4% experienced persistent hypopigmentation after full-face combination CO_2_/Er:YAG laser resurfacing.^32^

**Figure 5.**
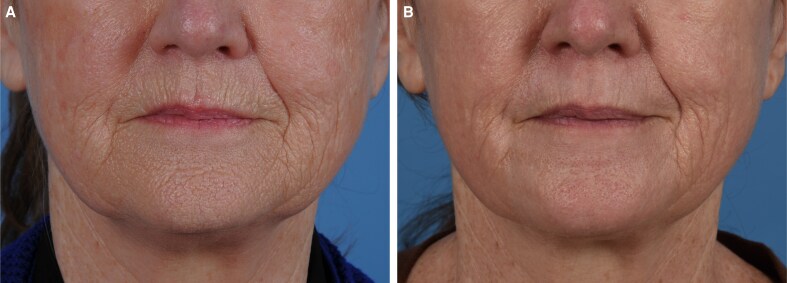
Case example of a 62-year-old female at (A) baseline and (B) 8 months following full-face 2940-nm Er:YAG resurfacing with hypopigmentation near the right oral commissure.

**Figure 6.**
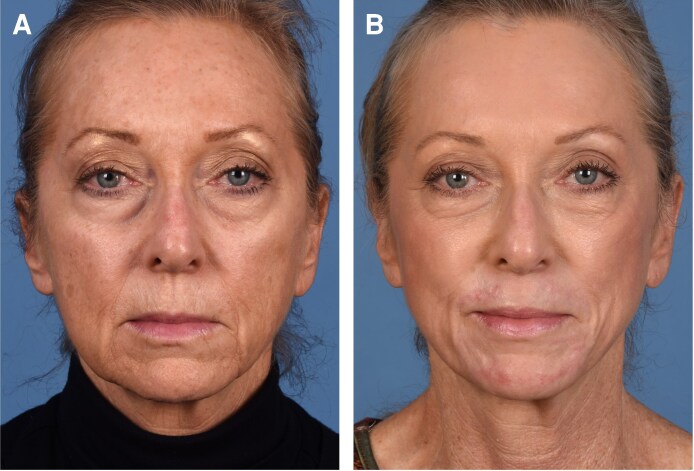
Case example of a 61-year-old female at (A) baseline and (B) 4 months following full-face 2940-nm Er:YAG resurfacing with perioral hypopigmentation and scarring.

Few studies have evaluated complication rates over a 10-year period, during which all laser and energy-based treatments were performed by the same operator.^28,32^ In this study, the overall complication rate was low (1.4% of procedures), although the true incidence is likely underestimated due to inconsistent documentation of mild or transient adverse events in chart review. Within the documented cases, hyperpigmentation was the most frequent complication, followed by hypopigmentation (Figure 5) and scarring (Figure 6), with the latter two occurring exclusively following ablative Er:YAG resurfacing, consistent with the known risk profile of ablative modalities.^28,33,34^ Treatment options for postprocedural scarring include further laser or energy-based procedures, intralesional 5-fluorouracil (5-FU), or corticosteroid injections.

Additional complications included neuropathic pain following a transient nerve injury, which was observed in 5 patients and presented approximately 2 weeks posttreatment. These cases were managed with gabapentin (300 mg 3 times daily) and alprazolam (0.25-0.5 mg 3 to 4 times daily) for 2 weeks. Herpes simplex virus (HSV) reactivation is another recognized risk for both ablative and nonablative procedures, especially with perioral treatment.^14,34,35^ In our cohort, one patient developed Bell's palsy following HSV reactivation despite being on antiviral prophylaxis, with eventual recovery over a 12-month period. Collectively, these findings underscore the importance of careful patient selection, pretreatment evaluation, device choice, and posttreatment care, along with a thorough understanding of device parameters to minimize injury to nontarget tissues where competing chromophore absorption or excessive heat diffusion increase risk.^28,34,36-38^

### Surgical Crossover

Prior studies indicate that patients who begin with nonsurgical facial rejuvenation may later pursue aesthetic surgery.^21-23^ For example, one plastic surgeon's experience reported 15.7% of patients who received injectables later underwent an operative procedure.^22^ Additionally, a broader national consumer survey found that nearly half of respondents preferred to receive both nonsurgical and surgical cosmetic procedures from the same provider.^23^ However, few studies have explored this trajectory specifically among patients treated with laser or energy-based modalities.

A key finding of this study is the high rate of same-practice surgical crossover (40.5%) following laser or energy-based treatments. This rate is substantially higher than previously reported regarding injectables and may be influenced by referral patterns and the academic practice setting, where patients seeking device-based rejuvenation may be more receptive to operative intervention. Notably, the resulting surgical procedures were broadly distributed across facial, breast, body contouring, and liposuction categories rather than being confined to the anatomic region initially treated. This cross-regional pattern suggests that laser and energy-based treatments may serve as an entry point into broader aesthetic care, facilitating ongoing patient engagement and refinement of aesthetic goals over time. Taken together, these findings support the role of nonsurgical rejuvenation, including lasers and energy-based technologies, as part of a continuum of aesthetic care rather than isolated treatment.

All laser and energy-based procedures reviewed in this manuscript were performed by the senior author, a board-certified plastic surgeon, allowing for a controlled and consistent treatment approach. In many practices, most of these procedures could be delegated to other providers to better utilize the plastic surgeon's time. That being said, the performance of these procedures by the surgeon allowed for increased patient–physician interaction and longitudinal engagement, which may have played a larger role in transitioning some of these patients to surgical procedures; however, this relationship could not be definitively established in the current study.

### Limitations

This study has several limitations. As a retrospective review, it is subject to incomplete electronic medical record documentation, particularly for transient or mild complications such as postinflammatory hyperpigmentation, which, although included, was likely underreported. Variability in patient follow-up intervals and self-reported concerns may also have introduced bias.

In addition, the absence of a control group limits comparisons to other treatment approaches, techniques, or providers. Patient motivations for treatment selection, escalation, or subsequent surgery were not captured, and the lack of patient-reported outcome measures further limits assessment of patient satisfaction and its relationship to treatment utilization or surgical decision-making. Furthermore, this study reflects the experience of a single plastic surgeon at a high-volume academic center with extensive expertise in laser and energy-based procedures, which may limit generalizability particularly given the relatively high use of ablative modalities. Observed changes in treatment patterns over time may reflect evolving surgeon experience and preference rather than solely patient-driven factors.

Finally, the department employs multiple licensed aestheticians who also administer laser and energy-based treatments; however, patients treated by these providers were not included in this analysis, which may introduce selection bias. Additionally, many patients may transition ongoing device-based maintenance care to these providers following initial treatment by the senior author. Such treatments were not captured in the dataset and may underestimate the total number of device-based encounters per patient. Patients initially treated by aestheticians who later underwent surgery with the senior author were also not reliably identified, potentially affecting estimates of surgical crossover.

Future studies exploring the patient characteristics, treatment preferences, patient-reported outcomes, and predictors of surgical crossover warrant further investigation.

## CONCLUSIONS

As the use of laser and energy-based devices continues to grow within aesthetic plastic surgery, understanding their safety profiles, utilization patterns, and role in longitudinal patient care is increasingly important. In this 10-year single-surgeon review, these devices demonstrated a low rate of documented complications (1.4%) and evolving utilization patterns, with increasing use of ablative modalities over time. A substantial proportion of patients (40.5%) underwent subsequent aesthetic surgery with the same surgeon, spanning a broad range of facial, breast, and body procedures. These findings highlight the role of laser and energy-based technologies not only as effective nonsurgical rejuvenation interventions but also as an important component of longitudinal patient engagement within a comprehensive aesthetic practice. Collectively, these modalities may help bridge nonsurgical and surgical care, supporting continuity of care within the broader continuum of aesthetic medicine.
